# Systematic review and meta-analysis of calculating degree of comorbidity of irritable bowel syndrome with migraine

**DOI:** 10.1186/s13030-023-00275-4

**Published:** 2023-06-08

**Authors:** Tatvan S. Todor, Shin Fukudo

**Affiliations:** 1 Department of Behavioral Medicine, Tohoku University Graduate School of Medicine, 2-1 Seiryo, Aoba, Sendai 980-8575 Japan; 2 Maastricht University, Maastricht, Netherlands

**Keywords:** Brain-gut interaction, Epidemiology, Irritable bowel syndrome, Meta-analysis, Migraine, Prevalence, Stress

## Abstract

**Background:**

Irritable bowel syndrome (IBS) and migraines are often comorbid each other. These disorders are likely to be bidirectionally linked through the gut-brain axis and share several underlying mechanisms including central nervous system sensitization. However, quantitative analysis of comorbidity was not reported enough. The aim of this systematic review and meta-analysis was to calculate the present degree of comorbidity of these two disorders.

**Methods:**

A literature search was performed searching for articles describing IBS or migraine patients with the same inverse comorbidity. Pooled odds ratios (ORs) or hazard ratios (HRs) with 95% confidence intervals (CIs) were then extracted. The total effect estimates were determined and presented by random effect forest plots for the group of articles with IBS patients with migraine and the group of articles on migraine sufferers with comorbid IBS separately. The average results of these plots were compared.

**Results:**

The literature search resulted in initial 358 articles and final 22 articles for the meta-analysis. The total OR values obtained were 2.09 [1.79 – 2.43] in IBS with comorbid migraine or headache, 2.51 [1.76 – 3.58] for migraineurs with comorbid IBS and an overall HR of 1 .62 [1.29 – 2.03] was found for cohort studies of migraine sufferers with comorbid IBS. A similar expression of a selection of other comorbidities was found in IBS and migraine patients, especially for depression and fibromyalgia a strong similarity was found in their expression rate.

**Conclusions:**

This systematic review with meta-analysis was the first to combine data on IBS patients with comorbid migraine and migraineurs with comorbid IBS. The fact that closely related existential rates were observed between these two groups should be used as motivation for future research to further investigate these disorders for why this similarity occurs. Mechanisms involved in central hypersensitivity such as genetic risk factors, mitochondrial dysfunction and microbiota are particularly good candidates. Experimental designs in which therapeutic methods for these conditions can be exchanged or combined may also lead to the discovery of more efficient treatment methods.

**Supplementary Information:**

The online version contains supplementary material available at 10.1186/s13030-023-00275-4.

## Introduction

Chronic pain disorders have a strong impact to impair an individual's quality of life. A large proportion of the global population is experiencing this impact as the prevalence of these disorders ranges from 10% to as much as 50% [1]. Irritable bowel syndrome (IBS) and migraine are conditions recognized under this category. With a worldwide prevalence of 4.1–11%, IBS is one of the most common disorders of gut-brain interaction (DGBI) [2]. Migraine also has a notable impact as it has been confirmed to be the 6th most debilitating condition based on the number of years lost due to disability [3]. In general, IBS and migraine are considered to be two separate clinical disorders due to their anatomically distant locations with associated local symptoms, thus dividing them into the gastrointestinal (GI) disorder or the neurological disorder [4]. This perspective may require change, as previous literature has pointed to similarities between the disorders in several aspects, supporting the idea of classifying them within an overarching disorder group [4–6]. Both IBS and migraine show similarity in prevalence, female dominance in patients, psychosomatic dysfunction, somatic pain symptoms, comorbidities and possible underlying biochemical mechanisms related to the development of central hypersensitivity [4, 7]. Numerous studies have reported that it is common for IBS patients to have comorbid migraine and vice versa that migraine patients exhibit IBS symptoms [8–12]. This supports the notion of these clinical manifestations coexisting rather than coincidentally occurring together.

A theoretical foundation that underlies their connection must first be established. The gut-brain axis has been discussed as the bridging link between these seemingly distinct GI and neurological disorders [13]. There exists a bidirectional relationship between the central nervous system (CNS) and the enteric nervous system (ENS) that innervates the GI tract [14]. Influence of communication along brain-gut axis includes not only the ENS and CNS but also the other parts of the autonomic nervous system (ANS), the immune system, the hypothalamic–pituitary–adrenal (HPA) axis, and the gut microbiota [15]. Through these systems, the brain can regulate gut functions related to sensory information processing, motility and secretion, and vice versa, the gut also influences brain functions such as cognition and pain perception [13, 14].

There are some resemblances in neural pathophysiology of IBS and migraine. IBS patients show borderline abnormality in electroencephalography [16]. Migraine also shows abnormal electroencephalogram in 61% of the patients [17]. These dysfunctions may be related to abnormality of some neurotransmitters. Serotonin (5-hydroxytryptamine: 5-HT) is one of candidates of responsible transmitters because 5-HT3 receptor antagonist is effective on patients with IBS with predominant diarrhea [18] and ones with migraine [19]. The other receptors including 5-HT1A, 5-HT1B/D, and 5-HT1F receptors have been shown to have a function leading to the reduction of pain [20, 21]. Several studies also indicated an abnormally increased activation of N-methyl-D-aspartate (NMDA) receptors in individuals suffering from IBS and migraine [22]. This could trigger hyperexcitability of central neurons involved in pain perception, which in turn may lead to the emergence of pain signals in inappropriate situations [7, 22]. These phenomena support rationale of calculating quantitative comorbidity of IBS and migraine.

Recognizing the coexistence of IBS and migraine could lead to considerations of distributing therapy targets across both the gut and brain. This in turn could lead to higher disease management efficiencies in the treatment-resistant patients [23]. To date, however, only unidirectional relationships have been described for these conditions in articles, such as IBS patients with comorbid migraine or migraineurs with comorbid IBS. This systematic review with meta-analysis aims to demonstrate an equal existential magnitude of comorbid migraine in IBS patients as comorbid IBS in migraineurs. We hypothesized that the prevalence, indicated in odds ratio (OR) with a confidence interval (CI) of 95%, of comorbid migraine in IBS patients would be close to equal to that of comorbid IBS in migraine patients. We also hypothesized that IBS and migraine share the resemble mechanism through other comorbidities.

## Methods

### Sources and search strategies

A literature search of articles reporting the simultaneous presence of both IBS and migraine in participants was conducted using literature databases PubMed, Cochrane Library, and Google Scholar. The search terms were "irritable bowel syndrome" and "migraine" of which MeSH terms and tiab-terms were specifically created for the PubMed search to have a wider reach (Supplementary Fig. 1). Our strategy included three rounds of selection, where firstly the filtering process of literature was based purely on the title, secondly the abstract and finally the articles underwent full review.

### Literature selection and data extraction

Based on the inclusion criteria set for this review, English-language articles with cohort, case–control or cross-sectional design were accepted. The desired publication date was after 2003 and the article quality score had to be at least 4, calculated as described by Zia et al., [2]. With regard to the sample characteristics, studies with a sample size of at least 50 per group were included. Participants with IBS and comorbid migraine or headache and migraineurs with comorbid IBS were eligible. Any subtype of IBS was allowed as well as migraine with or without aura. If the study analysed multiple DGBIs, only IBS data was used. For data extraction, it was important that OR or hazard ratio (HR) with 95% CI were reported along with the quantitative or percentage sample sizes of the cases and controls. Exclusion criteria allowed for the rejection of animal studies, studies with participants younger than 18 years of age, and studies that reported migraine by means of a mean somatic symptom score.

Data to be extracted from the literature were author name, date of publication, country of origin, study design, sample size, recruitment method, diagnosis method for IBS and migraine or headache, sample mean age, percentage of women and men, OR or HR with 95% CI and the extent to which other comorbidities occurred in percentages.

### Statistical analysis

Review Manager version 5.4 software was used for the current meta-analysis. Effect estimates were determined using generic inverse variance methodology yielding pooled OR with 95% CI and standard error for each study with a case–control or cross-sectional design. A random effect forest plot was selected to represent this data, if the I^2^ test indicated high heterogeneity between studies with a value greater than 75%. The HR with 95% CI values and standard errors was obtained from the cohort studies. Again, a separate random effect model was plotted in case the I^2^ test value was higher than 75%. In addition, funnel plots were created for all study groups to see if there was publication bias. Finally, to assess the extent to which the same comorbidities are present, a bar chart was made with the average rates of occurrence of various comorbidities in IBS and migraine patients. For each comorbid disorder, the overall presence was determined by averaging the incidence values of all studies that reported it. The strength of similarity was determined by the difference between the percentages of the IBS and migraine groups for each comorbid condition, with < 5% indicating strong similarity and < 10% indicating moderate similarity.

## Results

**Fig. 1:**
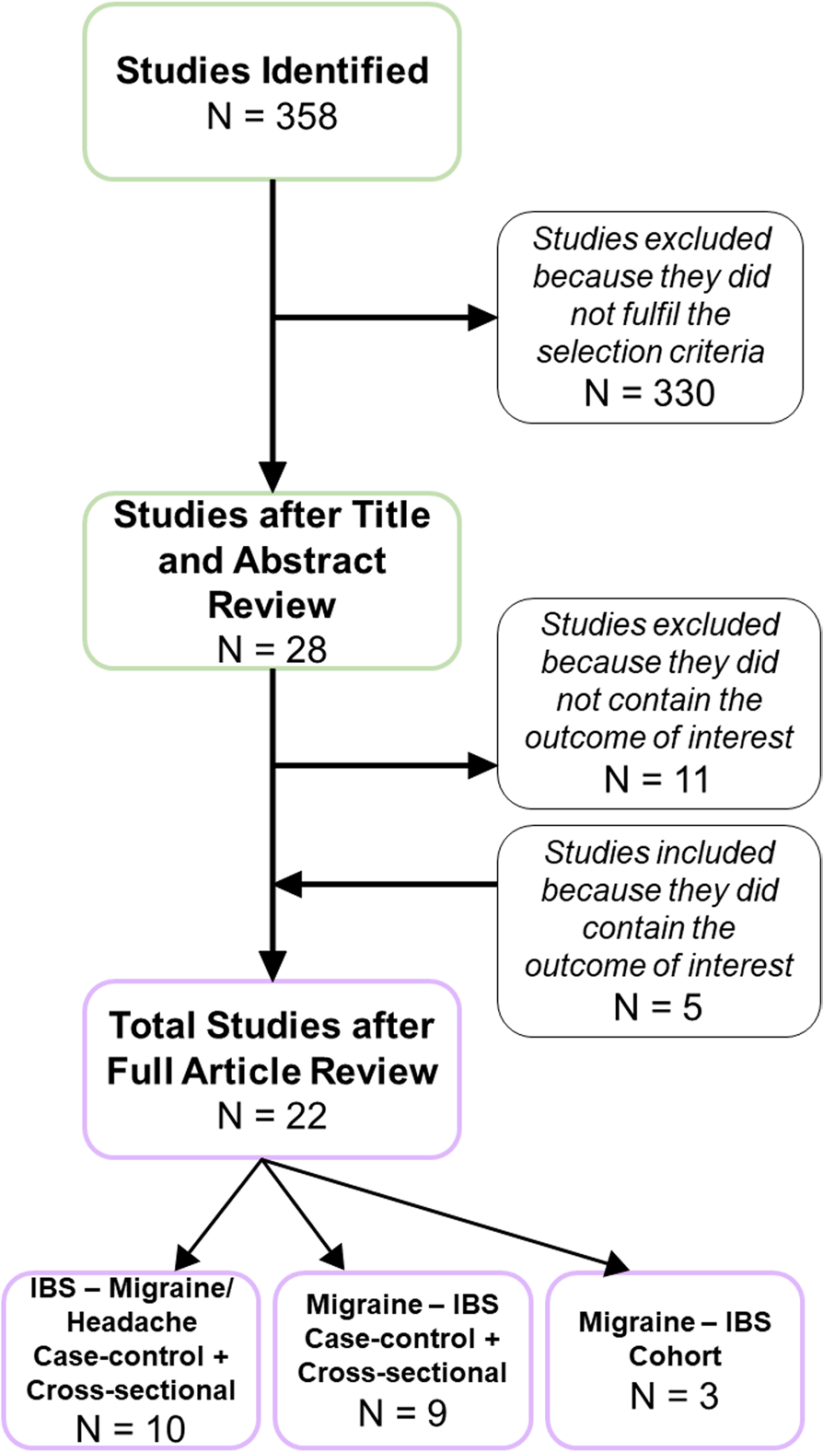
Flow diagram of literature selection procedure

The current systematic review with meta-analysis assessed the possible coexistence of IBS and migraine by observing an equal presence of comorbid migraine or headache in IBS and IBS comorbidity in migraineurs. A total of 358 articles emerged from the literature search. After the first two selection rounds based on title and abstract, 28 papers remained. These papers then underwent a full review. Subsequently, using the predetermined inclusion and exclusion criteria, the total number of papers ultimately used for analysis amounted 22 (Fig. 1) [9, 12, 24–43].

Clustering of the studies into different groups took place depending on pathological features and study design. The first group contained 10 articles exclusively with IBS patients who had comorbid migraine or headache [24–33]. In the second group, there were 9 articles on migraine sufferers with comorbid IBS [34–42]. These were all case–control or cross-sectional studies from which OR with 95% CI and standard errors were extracted and pooled (Table 1). Separately, the HR with 95% CI was extracted and pooled from 3 cohort studies of migraine sufferers who developed comorbid IBS [9, 12, 43].

**Table 1:** Overview of main characteristics and extracted data from articles included in this systematic review

**STUDY (Author + Year)**		**COUNTRY**			**STUDY DESIGN**	**N**					**RECRUITMENT**						**DIAGNOSIS IBS**		
**Migraineurs with IBS**																			
Wu (2017) [43]		Taiwan			Cohort	Total cases: 2859 Migraine: 2859 IBS: 239 Controls: 5718					Used data from the National Health Insurance Research Database (NHIRD) of Taiwan						Used disease history records with the International Classification of Disease, 9th Revision (ICD-9-CM), in which IBS has the following code: 564.1		
Lau (2014) [9]		Taiwan			Cohort	Total cases: 14,117 Migraine: 14,117 Controls: 56,468					Randomly selected 1 million people from the Taiwanese insurance claims database in the period of 1996 to 2010						Used the disease history as recorded in the International Classification of Disease, 9th Revision, Clinical Modification (ICD-9-CM), in which IBS has the following code: 564.1		
Penn (2019) [12]		Taiwan			Cohort	Total cases: 17,420 Migraine: 17,420 IBS: 3330 Controls: 69,680					Acquired data from the Longitudinal Health Insurance Database (LHID)						Used disease history records with the International Classification of Disease, 9th Revision (ICD-9-CM), in which IBS has the following code: 564.1		
Warren (2009) [34]		USA			Case–control	Total cases: 313 IBS: 86 Migraine: 112 Controls:313					Three ways. 1.Associations: the Interstitial Cystitis Association and the Interstitial Cystitis Network. 2.Professionals and support groups: urologists, gynaecologists, and regional IC/PBS support groups 3.Advertising: brochures, posters, national meetings, letters, newsletters, blast e-mails, and Website links. Controls were recruited by random digit dialling on a national scale						IBS diagnosis and onset was confirmed through a 6-step process using telephone interviews and medical record reviewing		
Martami (2017) [37]		Italy			Cross-sectional	Total cases: 1574 Migraine: 181 Headache78 Controls: 1315					Individuals that were referred to the Obesity Research Centre of Sina Hospital in the period from 2009 to 2016						Confirmed by a gastroenterology specialist. IBS was characterized according to ROME-III criteria		
Kim (2022) [41]		South Korea			Cross-sectional	Total cases: 781,115 IBS: 43,184 Migraine: 8438					Medical information reported to the Health Insurance Review & Assessment Service (HIRA) was used. Most Koreans are enrolled in this universal health insurance system. The dataset used random stratification based on 5-year interval ages and gender (HIRA-NPS-2018)						Confirmed using Code K58 of the Korean Standard Classification of Disease and Cause of Death-7 (KCD-7)		
Tietjen (2007) [35]		USA			Cross-sectional	Total cases: 171 Migraine: 171 IBS: 52 Controls: 104					Two different institutions: University of Toledo Medical Centre (Toledo, OH) and Duke University Medical Centre (Durham, NC)						Confirmed through a questionnaire that inquired the following self-reported physician-diagnosed conditions: “Have you ever been diagnosed by a doctor with IBS?”		
Grassini (2016) [42]		Sweden			Cross-sectional	Total cases: 151 Migraine: 151 IBS: 80 Controls: 3255					Acquisition of a representative sample of the general population from the county of Veasterbotten. Random selection from the population registry took place after stratification for sex and age						Used the Patient Health Questionnaire 15-Item Somatic Symptom Severity Scale (PHQ-15) and asking the question: “Have you been diagnosed with this disease by a physician?” The diseases included were fibromyalgia, IBS, CFS, exhaustion syndrome, depression		
Lankarani (2017) [40]		Iran			Cross-sectional	Total cases: 755 Migraine: 246 IBS: 184 Controls: 1609					Took place in Baladeha village near Kazerun, which is in the west of Fars province, Iran. Each individual older than 15 years was invited to participate in a medical interview at the health care center in this region						Used three-dimensional questionnaire which was completed during the physicians’ interview. The third dimension contained questions on gastrointestinal functional disorders symptoms in accordance with the ROME-III criteria		
Lee (2017) [39]		South Korea			Case–control	Total cases: 336 Migraine: 168 Headache168 Controls: 336					Clinical big data analytic solution Smart CDW from Hallym University Medical Centre (HUMC) was used of patients with common primary headaches (including migraines and TTH), and controls from January 2006 to August 2016 at the Chuncheon Sacred Heart Hospital of HUMC						Confirmed by physician after work-ups for patients who visited the gastroenterology centre more than 2 times consecutively		
Li (2017) [38]		China			Cross-sectional	Total cases: 1052 Migraine: 287 IBS: 312 Controls: 287					Used data of patients from the internal medicine and emergency departments of three hospitals (General Hospital of PLA, Rocket Army General Hospital, and the 316th Hospital of PLA) from June 2014 until 2016						IBS diagnosis was confirmed via the use of the ROME-III criteria		
McLean (2017) [36]		UK			Cross-sectional	Total cases: 1,468,404 Migraine: 9370 IBS: 52,333 Controls: 1,459,034					Data obtainment from the Primary Care Clinical Informatics Unit at the University of Aberdeen of patients that were permanently registered at one of 314 Scottish general practices on March 31, 2007						IBS diagnosis was confirmed through information in register		
**IBS patients with Migraine**																			
Ladabaum (2012) [30]		USA			Cross-sectional		Total cases: 141,295 IBS: 141,295 Migraine: 80,266 Control: 141,294				Recruitment from 1995 to 2005, of all individuals who were enrolled in KPNC. This population is demographically representative of the general population of northern California					Patients who had at least received 1 diagnosis from a medical doctor between 1995–2005			
Poitras (2007) [27]		Canada			Cross-sectional		Total cases: 167 IBS: 71 Migraine: 174 Control: 67				Patients followed by the gastroenterology department of the Hospital Saint-Luc, which is a tertiary care university hospital					E-mail questionnaire based on ROME-II criteria			
Vandvik (2004) [25]		Norway			Cross-sectional		Total cases: 208 IBS: 208 Migraine: 25 Control: 1240				Norwegian general practices. The study was executed during 2001 in nine practices in the county of Oppland					GPs reported on abdominal complaints using a paper questionnaire. Those who reported abdominal complaints within the past 3 months, were diagnosed according to the ROME-II criteria			
Cole (2006) [26]		USA			Cross-sectional		Total cases: 97,593 IBS: 97,593 Migraine: 6501 Control: 27,402				Data from eight different states with the largest concentration of health plan membership, primarily in mid/west and south/south-eastern United States					IBS diagnosis was established by using the ICD-9 CM. The corresponding code is 564.1			
**IBS patients with Headache**																			
Tuteja (2019) [32]	USA				Cross-sectional		Total cases: 413 IBS: 148 Control: 47					Data from list of GW Veterans from the Gulf War Registry of the Veterans Affairs Medical Centres in Salt Lake City, Utah and Gainesville (Florida). Data of 655 Veterans and 3.350 Veterans respectively. Other methods to recruit veterans was via advertisments			Previously validated Talley's Bowel Disease Questionnaire (BDQ) to assess current GI symptoms based on ROME-III criteria				
Przekop (2012) [29]	USA				Cross-sectional		Total cases: 598 IBS: 366 Headache: 3782 Control: 3213					Data drawn from the Biopsychosocial Religion and Health Study (BRHS). BRHS investigators randomly sampled individuals who participated in the Adventist Health Study 2 (AHS-2, 2002–2007)			Used the BRHS questionnaire. Physical symptom frequency in the past month was assessed by means of questions about how frequently participants experienced headache, indigestion, constipation, diarrhoea, and incontinence				
Whitehead (2007) [28]	USA				Cross-sectional		Total cases: 3724 IBS: 3153 Control: 3153					Used data of the Group Health Cooperative of Puget Sound (GHC). This is a large staff-model HMO that serves approximately 550.000 residents in Washington. GHC provides comprehensive health care primarily on a capitated basis			IBS was diagnosed using the ICD9-CM codes listed in the administrative database previously identified by the clinician at the time of clinic visit				
Tan (2003) [24]	Malaysia				Cross-sectional		Total cases: 533 IBS: 84 Headache: 228					Assessed the self-report questionnaires that were administered to a population of medical students from the Faculty of Medicine, University of Malaya			Questionnaire based on the Rome I criteria. It was defined as abdominal pain or discomfort for at least 3 months, which was relieved with defecation, associated with a change in frequency and consistency of stool				
Yanartas (2019) [33]	Turkey				Cross-sectional		Total cases: 207 IBS: 51 Headache 164 Control: 67					Gastroenterology and internal medicine outpatient clinic from March 2017 to September 2018 at Marmara University School of Medicine (Istanbul, Turkey)			IBS diagnosis was confirmed according to ROME-IV criteria				
Patel (2015) [31]	UK				Cross-sectional		Total cases: 840 IBS: 840 Headache 544 Control: 2137					Individuals who were newly referred from primary care to secondary care for investigation of GI symptoms. This took place at either McMaster University Medical Centre or St. Joseph’s Healthcare, both hospitals located in Hamilton (Ontario, Canada)			Data collected via the validated ROME-III diagnostic questionnaire for adult functional GI disorders. Through this the following information was recorded using a Likert scale: the frequency of individual lower GI symptoms, including lower abdominal pain or discomfort, stool frequency, stool consistency, bloating or abdominal distension, tenesmus and urgency				
**STUDY (Author + Year)**	**DIAGNOSIS MIGRAINE**			**DIAGNOSIS HEADACHE**					**MEAN AGE**						**QUALITY SCORE**				**HR/OR**
**Migraineurs with IBS**																			
Wu (2017) [43]	By means of the International Classification of Diseases 9th Revision (ICD-9-CM) with code 346 for migraine								Cases: 46.5 Controls: 46.1		71.4% Female 28.6% Male			9					HR: 1.58 (1.33–1.87)
Lau (2014) [9]	Disease history records with the International Classification of Disease, 9th Revision, Clinical Modification (ICD-9-CM), in which Migraine’s code is: 346								Total: 42.5 Migraine: 42.5 Controls: same migraine		72.6% Female, 27.4% Male			8					HR: 1.95 (1.75–2.18)
Penn (2019) [12]	Patients with a history of migraine (ICD-9-CM code 346)								Total cases: 44.5 Migraine: 44.5 Controls: 44.2		73.4% Female, 26.6% Male			10					HR: 1.36 (1.17 to 1.58)
Warren (2009) [34]	Through telephone interview which was used to identify 7 syndromes in total. This interview included expert consensus criteria for the following categories: CFS, IBS, panic, and migraine								Cases: 42.3 Controls: 42.9		100% Female			9					OR: 3.6 (2.3–5.6)
Martami (2017) [37]	Confirmed by a neurologist according to the international classification of headache disorders-III (ICHD-III-β)			TTH diagnosis was confirmed by a neurologist according to the international classification of headache disorders-III (ICHD-III-β)					Total cases: 37.44 Migraine: 38.39 Headache 41.08 Controls: 37.10		83.5% Female, 16.5% Male			8					OR: 4.90 (2.00–12.01)
Kim (2022) [41]	Used Code G43 of the Korean Standard Classification of Disease and Cause of Death-7 (KCD-7)								elderly (≥ 65 years) than in the adult group (≥ 20 and		50.1% Female, 49.9% Male			7					OR: 2.18 (2.04–2.33)
Tietjen (2007) [35]	Defined by the second International Classification of Headache Disorders (ICHD-II) criteria, through completion of the digital Headache Impact TestTM (HIT6)								Total cases: 39.1 Migraine: 37.6 Controls: 40.6		100% Female			9					OR: 2.7 (1.2–6.1)
Grassini (2016) [42]	Self-report on a received diagnosis of migraine by a physician								Total cases: 48.2 Migraine: 48.2 Controls: 51.4		64.6% Female, 35.4% Male			4					OR: 3.12 (1.60–6.06)
Lankarani (2017) [40]	Through the three-dimensional questionnaire completed during the physicians’ interview. The second dimension included questions on presence of headache symptoms based on criteria of International Headache Society								Total cases: 34.3		56.4% Female, 43.6% Male			8					OR: 3.43 (2.40–4.89)
Lee (2017) [39]	Reference to the International Classification of Headache Disorders (ICHD) second or third edition ( ICHD II or ICHD 3-beta)			Reference to the International Classification of Headache Disorders (ICHD) second or third edition ( ICHD II or ICHD 3-beta)					Range 19–80		83.9% Female, 16.1% Male			9					OR: 3.04 (0.50–18.35)
Li (2017) [38]	Based on the International Classification of Headache Disorders 3rd edition (ICHD-3-beta)								Total cases: 41.5 Migraine: 41.3 IBS: 40.2 Controls: 40.9		67.9% Female, 32.1% Male			8					OR: 1.07 (1.02–1.12),
McLean (2017) [36]	Based on whether patients had four or more anti-migraine prescriptions in the previous 12 months								Total cases: 48.8 Migraine: 50.5 Controls: 47.0		67.8% Female, 32.2% Male			7					OR: 2.22 (2.08–2.37)
**IBS patients with Migraine**																			
Ladabaum (2012) [30]	Based on patients who had at least received 1 diagnosis from a medical doctor between 1995–2005								Total cases: 53 IBS: 53 Control: 53	73.6% Female, 26.4% Male				9					OR: 2.31 (2.27–2.35)
Poitras (2007) [27]	E-mail questionnaire which also contained extra-GI related questions								Total cases: 43 IBS: 46.8 Control: 42.2	100% Female				9					OR: 2.4 (1.29–4.47)
Vandvik (2004) [25]	Self-administered questionnaires which had to be completed at the first visit								Total cases: 50.3 IBS: 50.3	67% Female, 33% Male				7					OR: 2 (1.2–3.5)
Cole (2006) [26]	Diagnosed by a physician, any past hospitalization associated migraine or outpatient prescriptions associated with anti-migraine drugs (e.g. ergot alkaloid or triptan). Both the diagnosis and the prescription criteria had to be fulfilled to be clasified withing the migraine group								aged 18 and older	75% Female, 25% Male				7					OR: 1.6 (1.4 – 1.7)
**IBS patients with Headache**																			
Tuteja (2019) [32]			Used the Somatic Symptom Checklist (SSC). The checklist was used to detect the following extra‐intestinal symptoms: **headache**, backache, wheeziness, insomnia, bad breath, fatigue, general stiff‐ ness, dizziness, weakness, sensitivity to hot and cold, palpitation, and tightness in chest					Range 32–78 Total cases: 47		10.5% Female, 89.5% Male				6				OR: 2.33 (1.36‐3.99)	
Przekop (2012) [29]			Used the BRHS questionnaire. Physical symptom frequency in the past month was assessed by means of questions about how frequently participants experienced **headache**, indigestion, constipation, diarrhea, and incontinence					Total cases: 63.1 IBS: 64.9 Control: 62.4		100% Female				4				OR: 0.52 (0.2–1.38)	
Whitehead (2007) [28]			Used the ICD9-CM codes listed in the administrative database previously identified by the clinician at the time of clinic visit					aged 18 and older		68.7% Female, 31.3% Male				9				OR: 2.40 (2.07–2.78)	
Tan (2003) [24]			Via questionnaire evaluating also other aspects of inquiry including alcohol intake, smoking, chili consumption, fibre intake, the presence anxiety, depression, insomnia, **headache**, and health-seeking behaviour					Total cases: 22		57% Female, 43% Male			5					OR: 1.7 (1.0–2.8)	
Yanartas (2019) [33]			Used the Bradford Somatic Inventory (BSI) which is a multi-ethnic inventory of functional somatic complaints associated with anxiety and depression. It measured a wide range of somatic symptoms during the previous month					Total cases: 35.1 IBS: 36 Control: 32.1		72.2% Female, 27.8% Male			10					OR: 2.21 (1.05–4.65)	
Patel (2015) [31]			Data obtained through the PHQ-12 questionnaire (excluded 3 GI questions). It asks about the presence of somatic symptoms over the last 4 weeks					Total cases: 43.3 IBS: 38.3 Control: 48.3		74.5% Female, 25.5% Male			7					OR: 2.58 (2.13–3.13)	

### Comparison of comorbidity rate in IBS and migraine patients

**Fig. 2:**
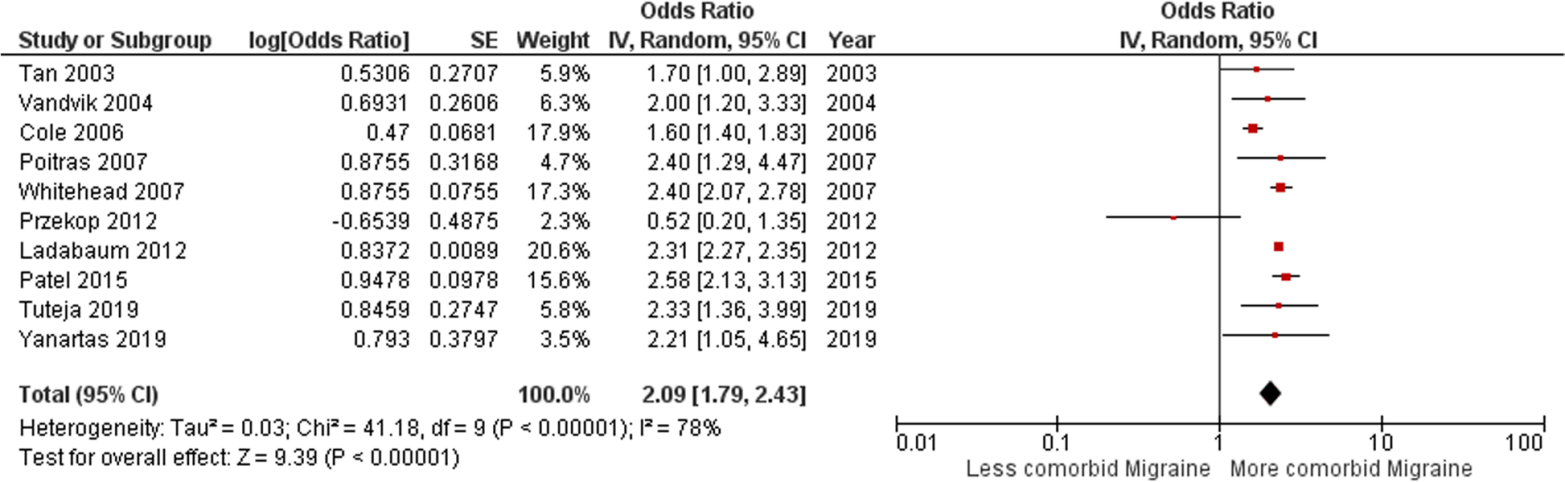
Forest plot for comorbid migraine or headache in IBS patients. Odds ratio (OR, red small box) and 95% confidence interval (CI, horizontal bar) in 10 case–control and cross-sectional studies were plotted. Black diamond showed calculated value of OR and 95%CI

**Fig. 3:**
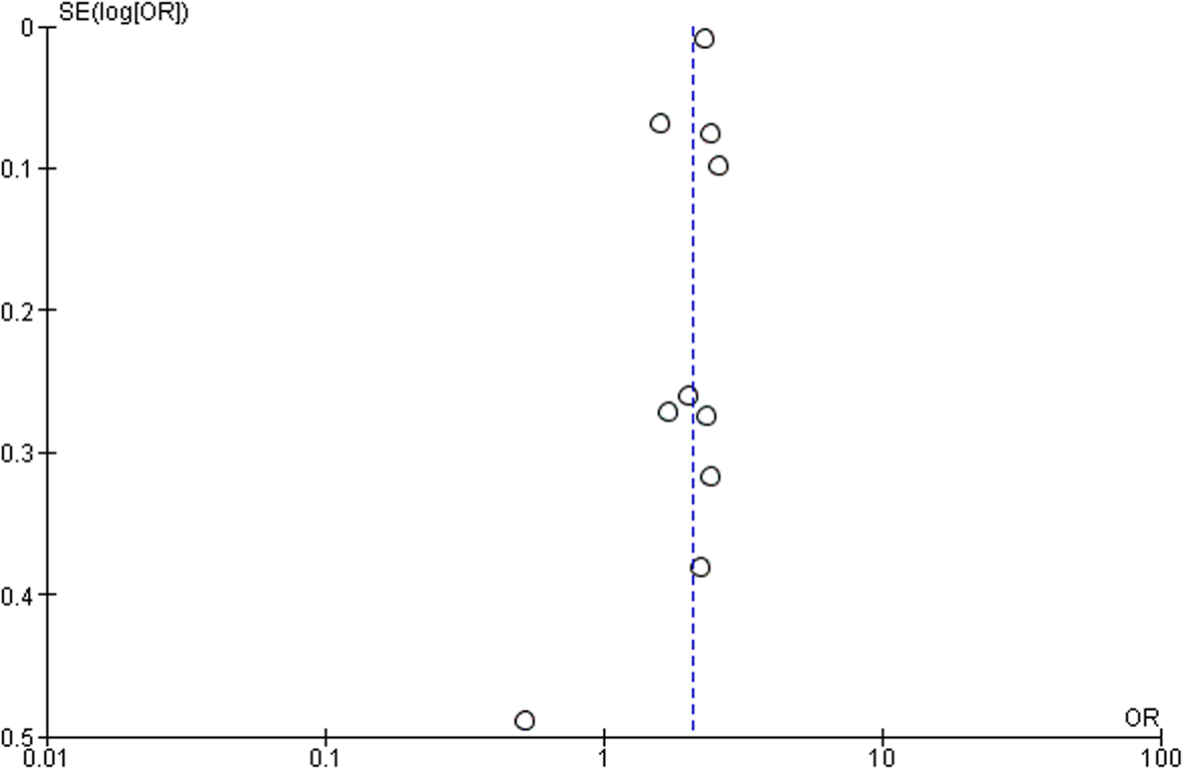
Funnel plot presenting association between IBS and migraine or headache comorbidity. Open circle showed 10 case–control and cross-sectional studies

The total OR with 95% CI resulting from the random effect forest plot analysis of IBS patient group with comorbid migraine or headache was 2.09 [1.79 – 2.43] (Fig. 2). This indicated a higher preference for comorbid migraine or headache in IBS subjects than not having these comorbidities. With the associated value of 78% for the I^2^ test, it can be confirmed that there was a high heterogeneity between these studies. This supported the choice for the random effect rather than the fixed effect model. However, for these articles within this category, the asymmetric funnel plot did indicate publication bias (Fig. 3) [24–33].

**Fig. 4:**
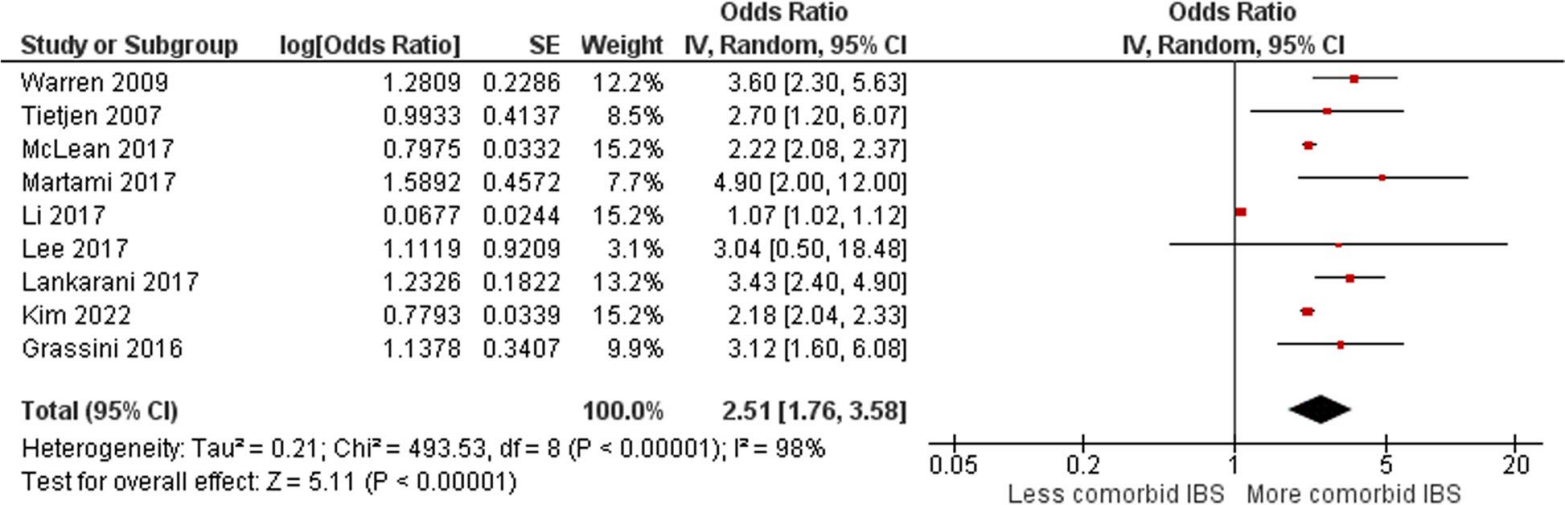
Forest plot for comorbid IBS in migraine patients. Odds ratio (OR, red small box) and 95% confidence interval (CI, horizontal bar) in 9 case–control and cross-sectional studies were plotted. Black diamond showed calculated value of OR and 95%CI

**Fig. 5:**
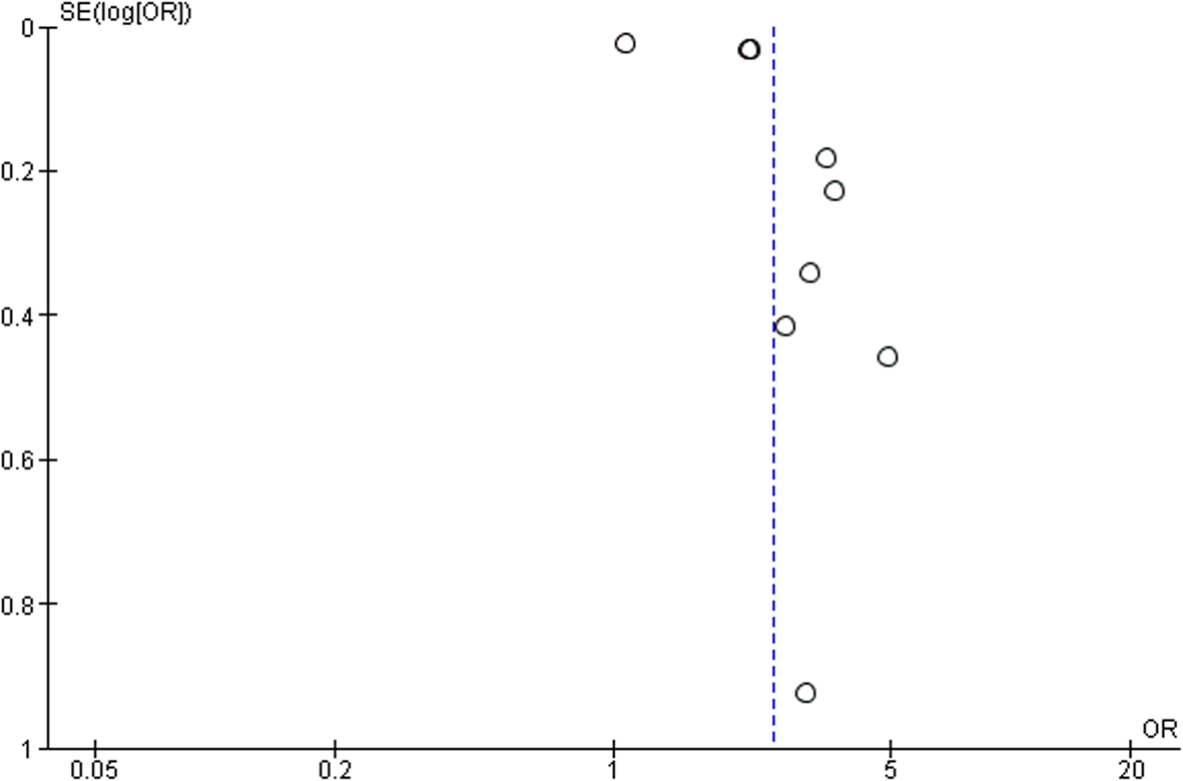
Funnel plot presenting association between migraine and IBS comorbidity Open circle showed 9 case–control and cross-sectional studies

For the category of migraine sufferers with comorbid IBS, the random effect model resulted in an overall OR value of 2.51 [1.76 – 3.58] (Fig. 4). This showed a stronger presence of comorbid IBS in migraine sufferers. Also for this model, with an I^2^ test value of 98%, a high heterogeneity between these studies was observed, making the random effect analysis the most optimal method. The respective funnel plot showed an even stronger asymmetry in this migraine with comorbid IBS group, which can be interpreted as strong publication bias (Fig. 5) [34–42].

### Development of comorbid IBS in longitudinal studies

**Fig. 6:**
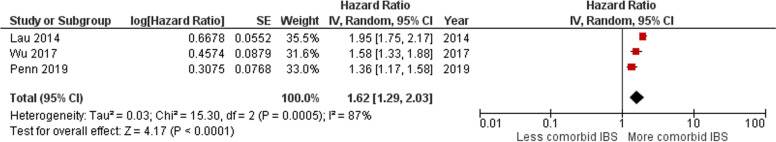
Forest plot for the development of comorbid IBS in migraine patients. Risk ratio (RR, red small box) and 95% confidence interval (CI, horizontal bar) in 3 cohort studies were plotted. Black diamond showed calculated value of RR and 95%CI

**Fig. 7:**
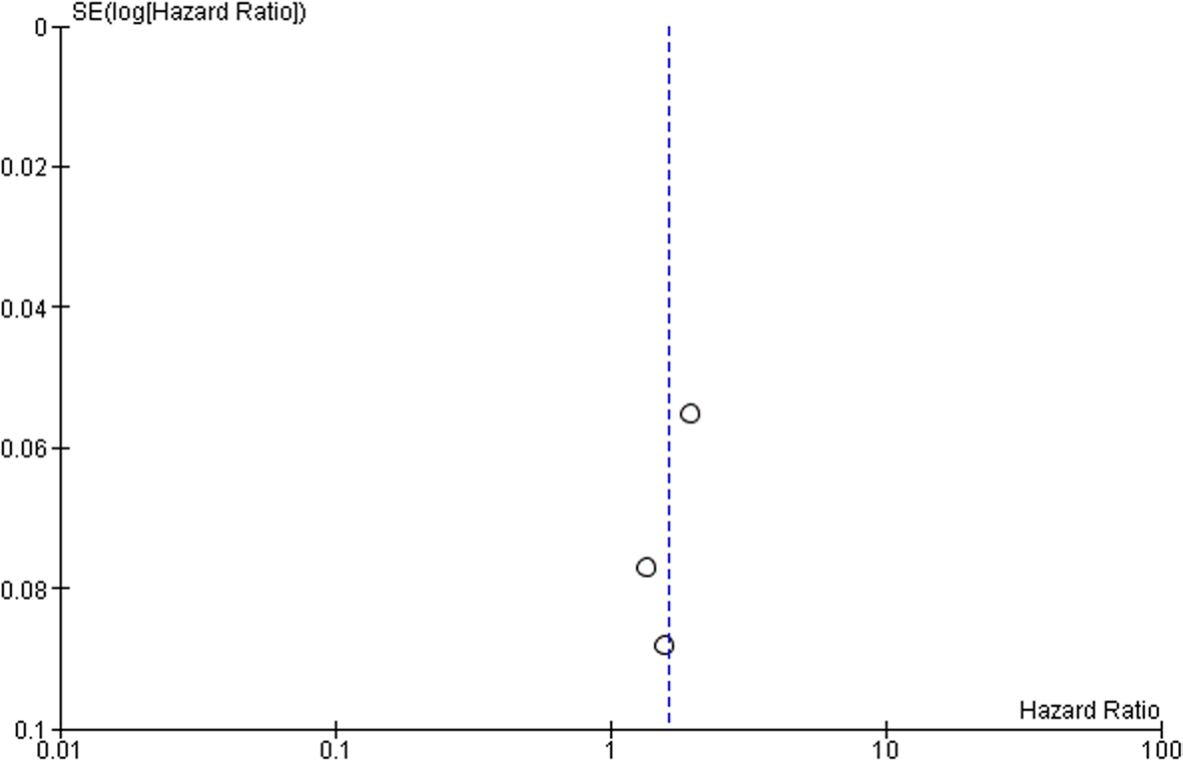
Funnel plot presenting association between migraine and IBS comorbidity development. Open circle showed 3 cohort studies

The random-effect forest plot of the cohort studies of migraineurs with comorbid IBS showed an overall HR with 95% CI of 1.62 [1.29 – 2.03] (Fig. 6). It can be argued from this that comorbid IBS is most likely to develop in migraine sufferers over time. High heterogeneity was observed between these cohort studies, as indicated by an I^2^ test result of 87%. For this reason, a random effect model was chosen. To check for publication bias, a funnel plot was again used and the asymmetry confirmed publication bias for these 3 studies (Fig. 7) [9, 12, 43].

### Prevalence comparison of other common comorbidities in IBS vs migraine patients

**Fig. 8:**
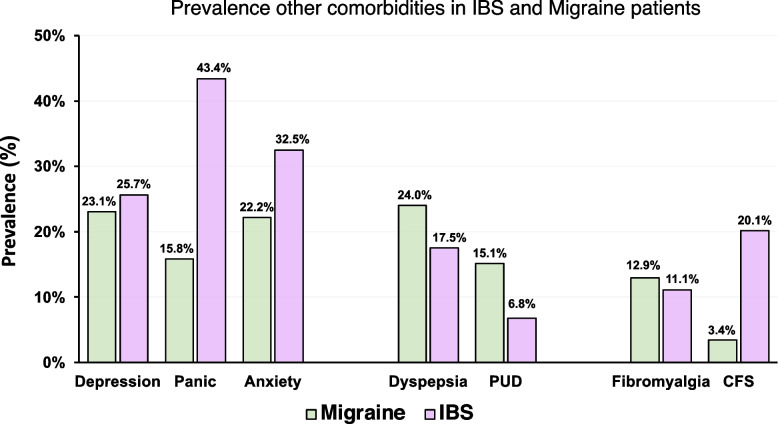
Prevalence of similar comorbidities in IBS and migraine patients. Prevalence (%) of comorbidity of depression, panic disorder, anxiety disorder, functional dyspepsia, peptic ulcer disease (PUD), fibromyalgia, and chronic fatigue syndrome (CFS) in migraine (green) and IBS (purple) patients were shown

To assess whether IBS and migraine may be part of a spectrum of centrally mediated hypersensitivity disorders, the possible presence of other comorbidities was determined. Depression, panic, anxiety, dyspepsia, peptic ulcer disease (PUD), fibromyalgia, and chronic fatigue syndrome (CFS) were all reported as comorbid in both IBS and migraine patients in multiple studies included in this systematic review (Additional file 2). In particular, depression (migraine – 23.07%, IBS – 25.66%) and fibromyalgia (migraine – 12.90%, IBS – 11.10%) showed strong similarity (< 5% difference) in their comorbid occurrence for both IBS as migraineurs. Also notable were the occurrence of dyspepsia (migraine – 23.99%, IBS – 17.48%) and PUD (migraine – 15.14%, IBS – 6.76%) with moderate similarity (< 10% difference) in their values between the IBS and migraine groups (Fig. 8) [9, 12, 24–43].

## Discussion

To our knowledge, this systematic review with meta-analysis was the first to analyze the coexistence of IBS and migraine by combining reciprocal data from IBS patients suffering from comorbid migraine with migraine patients who have comorbid IBS. A total of 22 studies were obtained, of which 10 contained IBS patients with comorbid migraine or headache and for the migraine group with comorbid IBS there were the remaining 12 studies [9, 12, 24–43]. The combined data provided a relatively large sample size of 286,993 IBS patients and 53,520 migraine patients. The results showed closely related OR values for case–control and cross-sectional studies reporting IBS and migraine comorbidity in both directions. These values were 2.09 [1.79 – 2.43] in IBS with comorbid migraine or headache and 2.51 [1.76 – 3.58] for migraineurs with comorbid IBS. The later value is comparable to OR 2.49 (95% CI, 2.22–2.78; *I*^2^, 42%) reported by another meta-analysis of prevalence of IBS in migraineurs [5]. With an overall HR of 1.62 [1.29 – 2.03], the cohort studies also showed evidence that migraineurs have a higher tendency to develop comorbid IBS, possibly supporting the claim of their coexistence. Finally, a similar expression of a selection of other comorbidities especially depression and fibromyalgia was found in IBS and migraine patients. Our study suggests that IBS and migraine have strong association with a comparable OR value greater than 2.

Several theories may explain this co-occurrence of IBS and migraines. These include mechanisms involved in central nervous system sensitization. Therefore, previous studies suggested that IBS and migraine could be good candidates for clinical reclassification as 'central hypersensitivity spectrum disorders' (CHSDs) [7, 23].

### Genes

The first theory explains this phenomenon through genetic influences. For IBS, genetic effects are expressed as a result of familial aggregation of risk genes [44]. Interesting candidates are genes involved in pain sensitization such as; 5-HT, substance P, nitric oxide (NO), noradrenaline, proteases, dynorphins and opiates [45]. IBS with predominant constipation has been found to have a significant association with alpha 1 and 2 variants of the adrenoceptor [46]. Another example more specific to abdominal pain symptoms is that there is a possible link to mutations in the SCN5A gene, which provide instructions for the construction of Na + channels in neuronal membranes. One study reported that 2% of IBS patients had a missense mutation on the G298S side of this gene [47]. A relationship between IBS and genes involved in the regulation of serotonin is often discussed in the literature. Several gene variants of 5-HT appear to play a role in the type and severity of symptoms [44, 48]. 5-HT3 appears to fulfill a function as a proalgesic, especially in IBS with predominant diarrhea. Another risk factor for IBS is the homozygous presence of the 5-HT2 allele [44]. Connections have also been made with serotonin regulation in migraine. Specifically, migraine with aura was associated with polymorphism in the serotonin transporter-linked promoter region (5-HTTLPR) [46, 48]. On the other hand, migraine without aura appeared to be influenced by the D4 dopamine receptor gene [44]. Lastly, nociceptive receptors such as transient receptor potential cation channel subfamily V member 1 (TrpV1) are also receiving attention as they may play a role in various functional pain disorders, including IBS and migraine [45]. Homozygous allelic variant rs222747 in TrpV1 was associated with higher glutamate activation, which in turn may be translated into increased cortical excitability in migraine sufferers [45]. Also, higher expression of TrpV1 at nerve fiber sites was correlated with visceral pain symptoms in IBS [49]. Shared gene analysis for IBS and migraine should be considered in the future.

### Mitochondria

Interestingly, the article by Meeus (2013) reported the influence of mitochondrial dysfunction in conditions such as fibromyalgia and CFS, both of which have been found in this review to be common comorbidities in both IBS and migraine patients [50]. It was described herein that oxidative and nitrosative stress-induced mitochondrial dysfunction could lead to decreased ATP availability in central neurons. As a downstream effect, NMDA receptor hypersensitivity arises in these cells. This results in long-term potentiation of pain signalling and eventual generalized central hypersensitivity to pain [50]. Not surprisingly, this relationship between mitochondrial dysfunction and an increased response to centrally mediated pain has also been reported in articles looking directly at IBS and migraine [51–53].

### Microbiota

The final theory to discuss regarding hypersensitivity in the central nervous system is due to the gut microbiota. IBS patients are known to have altered gut microbiota and their products [54]. Exacerbation of IBS symptoms is associated with rapid changes in gut microbiota with dynamic changes in the metabolites of neurotransmitters which are related to metabolic activity of gut microbiota [55]. Systematic review on gut microbiota disclosed decreased Faecalibacterium and Bifidobacterium as well as increased Lactobacillaceae, Bacteriodes, and Enterobacteriaceae in IBS patients [56]. Patients with migraine also have altered gut microbiota with increasing Firmicutes, especially the “unfriendly” Clostridium species and reduced *Faecalibacterium prausnitzii*, *Bifidobacterium adolescentis*, and *Methanobrevibacter smithii* with altered metabolites of neurotransmitters [57]. Especially concerning serotonin, fecal microbiome and their metabolome signatures reflect stress and serotonin metabolism in IBS patients [58]. Experiments conducted mainly in rodents have shown that the microbiota is involved in the development of not only IBS model [59] but also migraine model [60]. There was a study that extended nitroglycerin and antibiotics treatment in wild-type mice exacerbated the migraine phenotype through upregulation of tumor necrosis factor- ɑ (TNF- ɑ) [60] as well known in IBS patients [15]. Pain phenotypes in this migraine model were relieved by the administration of probiotic treatment [60] as previously reported in IBS patients [61]. More investigation to clarify underlying mechanisms on gut microbiota in IBS and migraine is warranted.

Concerning to the gut micro-organisms, a scientifically interesting question occurred to us. Infection of *Helicobacter pylori* (Hp) has strong associations with PUD and dyspepsia. As shown in Fig. 8 of this study, IBS and migraine patients had similar expression rates of PUD and dyspepsia. Does Hp relate to comorbidity of IBS and migraine? The first meta-analysis (2019) failed to establish a link between IBS and Hp infection [62]. The second systematic review and meta-analysis (2021) asserted Hp infection as a risk factor for the development of IBS and that therapeutic elimination of Hp reduces the developmental risk for IBS [63]. The third systematic review and meta-analysis (2022) showed lack of distinct association between IBS and Hp infection but positive association between IBS with diarrhea and Hp infection [64]. A meta-analysis pooling data from 5 case–control studies confirmed a higher frequency of Hp infections in migraine sufferers compared to controls [65]. This increased prevalence was again observed in a case–control study conducted in 2021, although migraine symptoms did not appear to be affected by Hp infections [66]. These studies suggest that the effects of Hp infection go beyond gastroduonenal pathologies. We previously reported that atrophic gastritis patients with positive anti-Hp antibody showed higher risk of depression than atrophic gastritis patients with negative anti-Hp antibody [67]. Interestingly, genome-wide association study of UK biobank revealed positive link between neural cell adhesion molecule (NCAM)-1 gene as a high risk loci for depression and IBS or Hp-relevant PUD/gastroesophageal reflux disease [68]. Large scale analyses including microorganisms, genes, and social environment should be performed in the near future.

This study has several limitations. First, this systematic review mainly included cross-sectional studies. Since these only provide insight into correlations between variables at a specific point in time, no conclusions can be made about any causal relationships between IBS and migraine. Since the mean pooled OR with 95% CI data was used as the main measure for answering the hypothesis, the possible influence of other factors cannot be denied and therefore coincidental co-existence of IBS and migraine cannot be completely rejected. It is therefore strongly recommended that future research should focus on conducting a systematic review with meta-analysis on this topic including cohort studies exclusively. Second, some studies in this review and meta-analysis used old diagnostic criteria. The switch from Rome III to Rome IV criteria has led to a lower prevalence of Rome IV-IBS than that of Rome III-IBS [69]. The newest diagnostic criteria of migraine are the 3rd edition of The International Classification of Headache Disorders [70]. However, headaches were also accepted as a measure of migraine, even though they are not clinically equivalent to migraine. Therefore, it should be considered that the study population was not homogenous. Third, we could not find several cohort studies with identifying migraine in IBS patients. This indicates the need for future research to perform a study design in which IBS patients are observed longitudinally, with the development of migraine being one of the variables of interest. Finally, the comparison of other comorbid disorders in IBS and migraine patients that we performed may be considered to be rough estimation. Although more detailed analysis on this paradigm was reported earlier [2], all studies in the past used independent criteria to identify the comorbid diseases. More accurate estimation is required in the future.

## Conclusion

This systematic review with meta-analysis was the first to combine data on IBS patients with comorbid migraine and migraineurs with comorbid IBS. The fact that closely related existential rates were observed between these two groups should be used as motivation for future research to further investigate these disorders for why this similarity occurs. Mechanisms involved in central hypersensitivity such as genetic risk factors, mitochondrial dysfunction and microbiota are particularly good candidates. Experimental designs in which therapeutic methods for these conditions can be exchanged or combined may also lead to the discovery of more efficient treatment methods.

## Supplementary Information

**Additional file 1: Supplementary Figure 1.** MeSH and tiab terms based on IBS and migraine or headache created for the literature search in PubMed.

**Additional file 2: Table S1.** Overview articles reporting other comorbidities in both IBS and AQ migraine patients.

## Abbreviations

ANS: Autonomic nervous system
CHSDs: Central hypersensitivity spectrum disorders
CNS: Central nervous system
CFS: Chronic fatigue syndrome
95% CI: 95% Confidence interval
DGBI: Disorders of gut-brain interaction
ENS: Enteric nervous system
GI: Gastrointestinal
HR: Hazard ratio
5-HTTLPR: 5-Hydroxytryptamine transporter-linked polymorphic region
HPA: Hypothalamic–pituitary–adrenal
IBS: Irritable bowel syndrome
NO: Nitric oxide
NMDA: N-methyl-D-aspartate
ORs: Odds ratios
PUD: Peptic ulcer disease
5-hydroxytryptamine: 5-HT: serotonin
TrpV1: Transient receptor potential cation channel subfamily V member 1
TNF-ɑ: Tumor necrosis factor- ɑ
