# Supplementary material for: Systematic review and meta-analysis of calculating degree of comorbidity of irritable bowel syndrome with migraine
Source: Biopsychosoc Med. 2023 Jun 8;17:22. doi: 10.1186/s13030-023-00275-4 (PMC10251688; doi:10.1186/s13030-023-00275-4)

**Supplementary Materials**

**Supplementary Figure 1. MeSH and tiab terms based on IBS and migraine or headache created for the literature search in PubMed.**


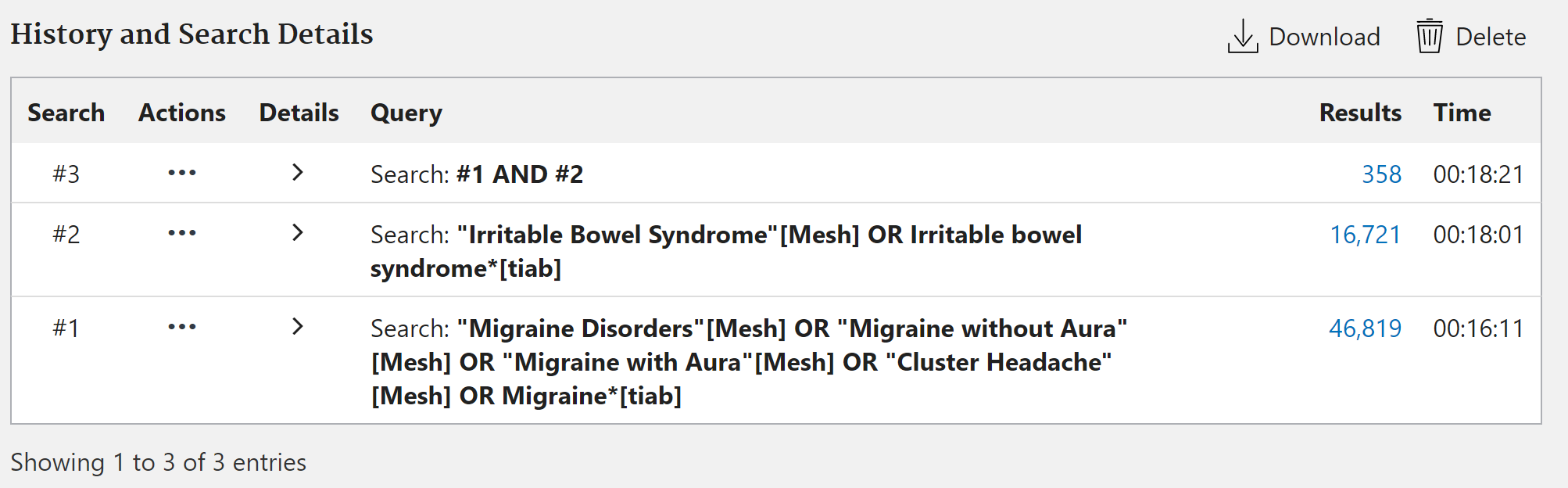

Supplement: Supplementary file 1 — Additional file 1: Supplementary Figure 1. MeSH and tiab terms based on IBS and migraine or headache created for the literature search in PubMed. [file 13030_2023_275_MOESM1_ESM.docx]
