# Supplementary material for: Systematic review and meta-analysis of calculating degree of comorbidity of irritable bowel syndrome with migraine
Source: Biopsychosoc Med. 2023 Jun 8;17:22. doi: 10.1186/s13030-023-00275-4 (PMC10251688; doi:10.1186/s13030-023-00275-4)
Supplement: Supplementary file 2 — Additional file 2: Table S1. Overview articles reporting other comorbidities in both IBS and AQ migraine patients. [file 13030_2023_275_MOESM2_ESM.pdf]

Table 2. Overview articles reporting other comorbidities in both IBS and migraine patients.

OTHER COMORBIDITIES	Depression	Panic	Anxiety	Epilepsy	Anorexia	Psychosis	Insomnia	Crohn’ s disease	Celiac disease	Dyspepsia	Endometriosis	IBD	Peptic ulcer disease	Interstitial cystitis	Fibromyalgia	Chronic Fatigue Syndrome	Chronic Pain
**Migraineurs with IBS**																	
Wu (2017)			■														
Lau (2014)	■		■												■		
Penn (2019)	■		■				■									■	
Warren (2009)	■	■									■				■	■	
Martami (2017)										■							
Kim (2022)										■		■	■				
Tietjen (2007)	■		■											■	■	■	

Cole (2006)	Vandvik (2004)	Poitras (2007)	Ladabaum (2012)	IBS patients with Migraine		McLean (2017)	Li (2017)	Lee (2017)	Lankarani (2017)	Grassini (2016)
					Depression					
					Panic					
					Anxiety					
					Epilepsy					
					Anorexia					
					Psychosis					
					Insomnia					
					Crohn’ s disease					
					Celiac disease					
					Dyspepsia					
					Endometriosis					
					IBD					
					Peptic ulcer disease					
					Interstitial cystitis					
					Fibromyalgia					
					Chronic Fatigue Syndrome					
					Chronic Pain					

Patel (2015)	Yanartas (2019)	Tan (2003)	Whitehead (2007)	Przekop (2012)	Tuteja (2019)	IBS patients with Headache	
							Depression
							Panic
							Anxiety
							Epilepsy
							Anorexia
							Psychosis
							Insomnia
							Crohn's disease
							Celiac disease
							Dyspepsia
							Endometriosis
							IBD
							Peptic ulcer disease
							Interstitial cystitis
							Fibromyalgia
							Chronic Fatigue Syndrome
							Chronic Pain
